# An azimuthally-modified linear phase grating: Generation of varied radial carpet beams over different diffraction orders with controlled intensity sharing among the generated beams

**DOI:** 10.1038/s41598-019-48757-2

**Published:** 2019-08-28

**Authors:** Saifollah Rasouli, Ali Mohammad Khazaei

**Affiliations:** 1 Department of Physics, Institute for Advanced Studies in Basic Sciences (IASBS), Zanjan, 45137-66731 Iran; 2 Optics Research Center, Institute for Advanced Studies in Basic Sciences (IASBS), Zanjan, 45137-66731 Iran

**Keywords:** Optical physics, Applied physics

## Abstract

Diffraction gratings are important optical components and are used in many areas of optics such as in spectroscopy. A diffraction grating is a periodic structure that splits and diffracts the impinging light beam into several beams travelling in different directions. The diffracted beams from a grating are commonly called diffraction orders. The directions of the diffraction orders depend on the grating period and the wavelength of the impinging light beam so that a grating can be used as a dispersive element. In the diffraction of a plane wave from a conventional grating, the intensities of diffracted beams decrease with increasing order of diffraction. Here, we introduce a new type of grating where in the diffraction of a plane wave, the intensity of a given higher order diffracted beam can be higher than the intensity of the lower orders. We construct these gratings by adding an azimuthal periodic dependency to the argument of the transmission function of a linear phase grating that has a sinusoidal profile and we call them azimuthally-modified linear phase gratings (AMLPGs). In this work, in addition to introducing AMLPGs, we present the generation of varied radial carpet beams over different diffraction orders of an AMLPG with controlled intensity sharing among the generated beams. A radial carpet beam is generated in the diffraction of a plane wave from a radial phase grating. We show that for a given value of the phase amplitude over the host linear phase grating, one of the diffraction orders is predominant and by increasing the value of the phase amplitude, the intensity sharing changes in favor of the higher orders. The theory of the work and experimental results are presented. In comparison with the diffraction of a plane wave from radial phase gratings, the use of AMLPGs provides high contrast diffraction patterns and presents varied radial carpet beams over the different diffraction orders of the host linear phase grating. The resulting patterns over different diffraction orders are specified and their differences are determined. The diffraction grating introduced with controlled intensity sharing among different diffraction orders might find wide applications in many areas of optics such as optical switches. We show that AMLPG-based radial carpet beams can be engineered in which they acquire sheet-like spokes. This feature nominates them for potential applications in light sheet microscopy. In addition, a detailed analysis of the multiplication of the diffraction pattern of an AMLPG by the 2D structure of a spatial light modulator is presented. The presented theory is confirmed by respective experiments.

## Introduction

A conventional optical diffraction grating, say linear grating, is a periodic structure in the Cartesian coordinates. In the diffraction of a plane wave from a linear grating, the beam is split and diffracted into several beams travelling in different directions. These diffracted beams are known as diffraction orders. For the conventional gratings, the intensities of diffraction orders decrease with increasing the number of order of diffraction. In the near-field regime, superposition of diffraction orders form self-images and sub-images of the grating’s structure at certain propagation distances. This effect is known as Talbot effect^1^. In addition, the intensity pattern over a plane includes the propagation axis and the grating vector (say the longitudinal plane) that is called the Talbot carpet^2^. In the far-field regime, the diffraction orders appear as the impulses of the Fourier transform of the grating’s transmission function.

The linear gratings have numerous applications in optics and other areas of sciences and technologies such as in astronomical spectroscopy, moiré fringe technique including moiré deflectometry and moiré topography^3^, interferometry^4–6^, lithography^7,8^, strain and stress analysis^9^, displacement measurement^10^, optical alignment technique, color printing^11^, and 3D displays^12,13^. In recent two to three decades amplitude and phase gratings with topological defects have found serious applications in generating vortex beams and/or characterizing such beams^14–21^. The use of phase hologram gratings instead of the conventional one has some advantages^22–25^. For instance, a computer generated hologram phase grating provides the implemention of a desired phase map on the incident wave in a controlled way^26^.

Another specific type of grating is radial grating. A radial grating has a periodic structure in the azimuthal direction in polar coordinates. Unlike conventional gratings, there is no diffraction order in diffraction from a radial grating. However, the diffraction of a plane or Gaussian wave from radial gratings shows other interesting features. On illuminating an amplitude radial grating by a spatially coherent light beam the Talbot carpet is formed on transverse planes^27^. The conventional Talbot carpet is formed on the longitudinal plane when a grating is illuminated by a plane beam^2,28,29^. It has also been shown that the plane boundaries between the optical regimes including the geometric shadow and near-field and far-field diffraction regimes have acquired curvature^27^. The diffraction of a vortex beam from an amplitude radial grating presents a simple way for measuring the topological charge alteration^30^. That study also presents a Poisson-Arago spot-like effect on the optical axis when an amplitude grating is illuminated by a vortex beam in which the number of grating spokes is equal to the topological charge of the incident beam. More precisely, a needle-like optical beam forms along the optical axis in far-field diffraction. The diffraction of a plane wave from a radial phase grating was also theoretically and experimentally investigated. It was shown that the diffraction of a plane wave from radial phase gratings is a simple way to generate radial carpet beams that are nondiffracting, accelerating, and shape preserving^31^. These beams have unprecedented patterns that are shape-invariant during propagation. The patterns can be easily turned into tunable 2D optical lattices. We also proposed a new solution for the paraxial Helmholtz equation and introduced combined half-integer Bessel-like beams^32^. It was shown that a huge family of beams can be produced in diffraction from designed radial structures and not necessarily from radial gratings. In that work spatially asymmetric, radial carpet, petallike, and twisted-intensity ringlike vortex beams were introduced through detailed theory and suitable practical approaches were proposed to generate each of them.

In this work, we integrate the features of linear and radial gratings to introduce a new grating that, in addition to the properties of linear and radial gratings, has additional properties. We construct this type of grating by adding an azimuthal periodic dependency to the argument of the transmission function of a linear phase grating that has a sinusoidal profile and we call them azimuthally-modified linear phase gratings (AMLPGs). In the diffraction of a plane wave from an AMLPG, we observe different diffraction orders in which the pattern of each order is very similar (but not exactly equal) to the diffraction pattern of a radial grating. Unlike the conventional gratings, here the intensity of a given higher order diffracted beam can be higher than the intensities of the lower orders. We show that the intensity sharing among different diffraction orders of an AMLPG can be adjusted by the value of the phase amplitude of the host linear grating. This kind of grating might find wide applications in many areas of optics such as optical switches. The diffraction of a plane wave from AMLPGs is formulated and using a spatial light modulator (SLM) the respective experimental works are presented. In comparing with the diffraction of a plane wave from radial phase gratings^31^ the use of AMLPGs provides a set of high contrast and varied radial carpet patterns over the different diffraction orders of the host linear phase grating. Since an SLM has a two dimensional periodic structure it multiples the diffraction pattern of the AMLPG. A detailed formulation is also presented and the theoretical predictions are verified by experiments.

It is worth noting that, as the transmittance of an AMLPG is not the product of the transmittances of the radial and linear gratings, the diffraction pattern from an AMLPG is not the convolution of the diffraction patterns of radial and linear gratings. However, the transmittance of an AMLPG imposed on an SLM is the product of an ideal AMLPG and the two-dimensional periodic structure of the SLM, therefore the resulting diffraction pattern is the convolution of the diffraction pattern of the ideal AMLPG and the two dimensional impulses of the SLM.

## Results

### Formulation of plane wave diffraction from an AMLPG

**Figure 1:**
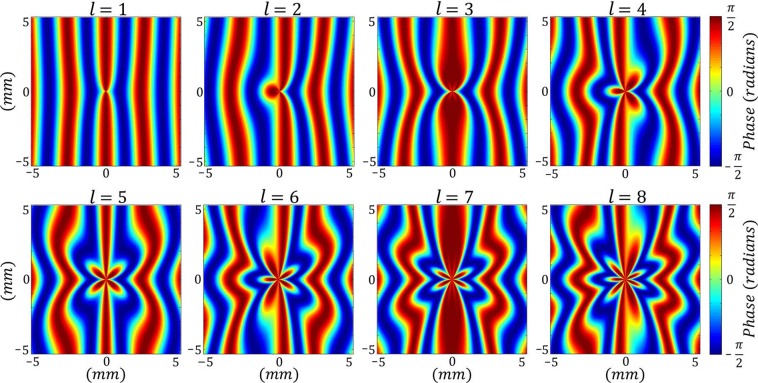
Illustration of eight typical AMLPGs with $d=3\,{\rm{mm}}$, $\gamma =\pi /2$, and different values of *l*.

Here, we formulate the diffraction of a plane wave from an AMLPG. When the phase structure of a linear phase grating with a sinusoidal profile hosting an additional radial phase grating, it can be considered as “azimuthally-modified linear phase grating (AMLPG)”. Some AMLPGs with almost sinusoidal phase profiles are shown in Fig. 1. Since a radial phase grating is periodic in the azimuthal direction and has a phase singularity at the origin, an AMLPG has also the same property.

We introduce an AMLPG with the following transmission function:

1 $$T(\rho ,\theta )=\exp [i\gamma \,\cos (\frac{2\pi }{d}\rho \,\cos \,\theta +\,\cos \,l\theta )],$$

where *γ*, *l*, *d*, and $(\rho ,\theta )$ are the amplitude of the phase variation, the number of spokes of the radial part of the grating, the period of the linear part of the grating, and the polar coordinates in the input plane, respectively.

As is apparent from Eq. 1, the transmission function of the AMLPG is not the product of the transmission functions of two distinct radial and linear gratings^33^. Therefore the diffraction pattern from an AMLPG is not the convolution of the diffraction patterns of the radial and linear gratings. Below we formulate the diffraction of a plane wave from an AMLPG.

Using the Jacobi-Anger identity^34^

2 $${{\rm{e}}}^{i\gamma \cos \theta }=\mathop{\sum }\limits_{s=-\infty }^{s=+\infty }\,{(i)}^{s}{J}_{s}(\gamma ){{\rm{e}}}^{is\theta },$$

where *J*_*s*_ is the *s*–th Bessel function of the first kind, the grating’s transmission function, Eq. 1, can be rewritten in the following form:

3 $$T(\rho ,\theta )=\mathop{\sum }\limits_{s=-\infty }^{s=+\infty }\,{(i)}^{s}{J}_{s}(\gamma )exp[is(\frac{2\pi }{d}\rho \,\cos \,\theta +\,\cos \,l\theta )].$$

By illuminating this phase structure with a plane wave, the complex amplitude of the light field after the structure is given by

4 $$U(r,\phi ,z)={h}_{0}\,{{\rm{e}}}^{i\alpha {r}^{2}}\,{\int }_{0}^{\infty }\,{\int }_{0}^{2\pi }\,T(\rho ,\theta ){{\rm{e}}}^{i\alpha {\rho }^{2}}{{\rm{e}}}^{-2i\alpha \rho r\cos (\theta -\phi )}\rho \,d\rho \,d\theta ,$$

where ${h}_{0}=\frac{{e}^{ikz}}{i\lambda z}$ and $\alpha =\frac{\pi }{\lambda z}$, in which *λ* is the wavelength of the light beam, $k=\frac{2\pi }{\lambda }$ is the wave-number, and $(r,\phi )$ are the polar coordinates on the output plane.

By substituting Eq. 3 in Eq. 4, we have

5 $$\begin{array}{ccc}U(r,\phi ,z) & = & {h}_{0}\,{{\rm{e}}}^{i\alpha {r}^{2}}\,{\int }_{0}^{{\rm{\infty }}}\,{\int }_{0}^{2\pi }\,\mathop{\sum }\limits_{s=-{\rm{\infty }}}^{+{\rm{\infty }}}\,{(i)}^{s}{J}_{s}(\gamma ){e}^{[is(\frac{2\pi }{d}\rho \cos \theta +\cos l\theta )]}\\  &  & \times \,{{\rm{e}}}^{i\alpha {\rho }^{2}}{{\rm{e}}}^{-2i\alpha r\rho \cos (\theta -\phi )}\rho \,d\rho d\theta \\  & = & {h}_{0}\,{{\rm{e}}}^{i\alpha {r}^{2}}\,{\int }_{0}^{{\rm{\infty }}}\,{\int }_{0}^{2\pi }\,\mathop{\sum }\limits_{s=-{\rm{\infty }}}^{+{\rm{\infty }}}\,{(i)}^{s}{J}_{s}(\gamma ){{\rm{e}}}^{i\alpha {\rho }^{2}}{{\rm{e}}}^{-2i\alpha \rho r\sin \phi sin\theta }\\  &  & \times \,{{\rm{e}}}^{-2i\alpha \rho \cos \theta (r\cos \phi -\frac{s\lambda z}{d})}{{\rm{e}}}^{is\cos 1\theta }\rho \,d\rho d\theta .\end{array}$$

Equation 5 can be solved respect to the azimuthal variable. First we use the following variable transformations in the output plane:

6 $$r\,\cos \,\phi -\frac{s\lambda z}{d}={r}_{s}\,\cos \,{\phi }_{s},\,r\,\sin \,\phi ={r}_{s}\,\sin \,{\phi }_{s},$$

where

7 $$\begin{array}{rcl}{r}_{s} & = & \sqrt{{r}^{2}+{(\frac{s\lambda z}{d})}^{2}-2\frac{s\lambda z}{d}r\,\cos \,\phi },\\ {\phi }_{s} & = & {\tan }^{-1}(\frac{r\,\sin \,\phi }{r\,\cos \,\phi -\frac{s\lambda z}{d}}),\\ s & = & 0,\pm \,1,\pm \,2,\ldots \end{array}$$

and rewrite Eq. 5 in the following form:

8 $$U(r,\phi ,z)={h}_{0}\,{{\rm{e}}}^{i\alpha {r}^{2}}\,{\int }_{0}^{{\rm{\infty }}}\,\mathop{\sum }\limits_{s=-{\rm{\infty }}}^{+{\rm{\infty }}}\,{(i)}^{s}{J}_{s}(\gamma ){{\rm{\Theta }}}_{s\cos 1\theta }{{\rm{e}}}^{i\alpha {\rho }^{2}}\rho \,d\rho ,$$

in which

9 $${{\rm{\Theta }}}_{s\cos {\rm{1}}\theta }={\int }_{0}^{2\pi }\,{{\rm{e}}}^{-2i\alpha \rho {r}_{s}\cos (\theta -{\phi }_{s})}\,{{\rm{e}}}^{is\cos {\rm{1}}\theta }d\theta \mathrm{.}$$

Now using Eqs 2 and 9 reduces to the following form:

10 $$\begin{array}{rcl}{{\rm{\Theta }}}_{s\cos {\rm{1}}\theta } & = & {\int }_{0}^{2\pi }\,\mathop{\sum }\limits_{p=-\infty }^{+\infty }\,{(-i)}^{p}{J}_{p}\mathrm{(2}\alpha \rho {r}_{s}){{\rm{e}}}^{ip(\theta -{\phi }_{s})}\,\mathop{\sum }\limits_{q=-\infty }^{+\infty }\,{(i)}^{q}{J}_{q}(s){{\rm{e}}}^{iql\theta }\,d\theta \\  & = & \mathop{\sum }\limits_{p=-\infty }^{+\infty }\,\mathop{\sum }\limits_{q=-\infty }^{+\infty }\,{\int }_{0}^{2\pi }\,{(-i)}^{p}{(i)}^{q}{J}_{p}\mathrm{(2}\alpha \rho {r}_{s}){J}_{q}(s){{\rm{e}}}^{i(p+ql)\theta }{{\rm{e}}}^{-ip{\phi }_{s}}d\theta ,\end{array}$$

and using the following identity:

11 $${\int }_{0}^{2\pi }\,{e}^{i(p+ql)\theta }d\theta =2\pi {\delta }_{(p,-ql)},$$

we have

12 $${{\rm{\Theta }}}_{s\cos {\rm{1}}\theta }=2\pi \,\mathop{\sum }\limits_{q=-\infty }^{+\infty }\,{(-i)}^{-ql}{(i)}^{q}{J}_{-ql}\mathrm{(2}\alpha \rho {r}_{s}){J}_{q}(s){{\rm{e}}}^{iql{\phi }_{s}}\mathrm{.}$$

As ${J}_{-n}(x)={(-1)}^{n}{J}_{n}(x)$, we have

13 $${{\rm{\Theta }}}_{s\cos {\rm{1}}\theta }=2\pi \,\mathop{\sum }\limits_{q=-\infty }^{+\infty }\,{(-i)}^{ql}{(i)}^{q}{J}_{ql}\mathrm{(2}\alpha \rho {r}_{s}){J}_{q}(s){{\rm{e}}}^{iql{\phi }_{s}}\mathrm{.}$$

By substituting Eq. 13 in Eq. 8 we have

14 $$\begin{array}{rcl}U(r,\phi ,z) & = & 2\pi {h}_{0}\,{{\rm{e}}}^{i\alpha {r}^{2}}\,\mathop{\sum }\limits_{s=-\infty }^{+\infty }\,\mathop{\sum }\limits_{q=-\infty }^{+\infty }\,{(i)}^{s}{(i)}^{q}{(-i)}^{ql}{J}_{s}(\gamma ){J}_{q}(s){{\rm{e}}}^{iql{\phi }_{s}}\\  &  & \times \,\,{\int }_{0}^{\infty }\,{J}_{ql}\mathrm{(2}\alpha \rho {r}_{s}){{\rm{e}}}^{i\alpha {\rho }^{2}}\,\rho \,d\rho .\end{array}$$

Now using the following integral identity^35^:

15 $${\int }_{0}^{\infty }\,{J}_{v}(b\rho ){{\rm{e}}}^{i\alpha {\rho }^{2}}\rho \,d\rho =\frac{b}{8}(\frac{\sqrt{\pi }}{{\alpha }^{\frac{3}{2}}}){e}^{-i(\frac{{b}^{2}}{8\alpha }-\frac{v\pi }{4})}[{J}_{\frac{v+1}{2}}(\frac{{b}^{2}}{8\alpha })+i\,{J}_{\frac{v-1}{2}}(\frac{{b}^{2}}{8\alpha })],$$

the resulting light field can be written in the following form:

16 $$\begin{array}{rcl}U(r,\phi ,z) & = & {{\rm{e}}}^{ikz}{{\rm{e}}}^{i\alpha {r}^{2}}\{\mathop{\sum }\limits_{s=-\infty }^{+\infty }\,{(i)}^{s}{J}_{s}(\gamma ){J}_{0}(s){e}^{-i\alpha {r}_{s}^{2}}\\  &  & +\,\mathop{\sum }\limits_{s=-\infty }^{+\infty }\,\mathop{\sum }\limits_{q=1}^{+\infty }\,{(i)}^{s}{(i)}^{-q(\frac{l}{2}-1)-1}{J}_{s}(\gamma ){J}_{q}(s){r}_{s}(\frac{\pi }{\sqrt{\lambda z}}){e}^{\frac{-i\alpha {r}_{s}^{2}}{2}}\\  &  & \times \,[{J}_{\frac{ql+1}{2}}(\frac{\alpha {r}_{s}^{2}}{2})+i\,{J}_{\frac{ql-1}{2}}(\frac{\alpha {r}_{s}^{2}}{2})]\,\cos (ql{\phi }_{s})\}.\end{array}$$

Here *s* shows the number of the diffraction order of the AMLPG, and the corresponding light field is

17 $$\begin{array}{rcl}{U}_{s}({r}_{s},{\phi }_{s},z) & = & {{\rm{e}}}^{ikz}{{\rm{e}}}^{i\alpha {r}^{2}}\{{(i)}^{s}{J}_{s}(\gamma ){J}_{0}(s){e}^{-i\alpha {r}_{s}^{2}}\\  &  & +\,\mathop{\sum }\limits_{q=1}^{+\infty }\,{(i)}^{s}{(i)}^{-q(\frac{l}{2}-1)-1}{J}_{s}(\gamma ){J}_{q}(s){r}_{s}(\frac{\pi }{\sqrt{\lambda z}}){e}^{\frac{-i\alpha {r}_{s}^{2}}{2}}\\  &  & \times \,[{J}_{\frac{ql+1}{2}}(\frac{\alpha {r}_{s}^{2}}{2})+i\,{J}_{\frac{ql-1}{2}}(\frac{\alpha {r}_{s}^{2}}{2})]\,\cos (ql{\phi }_{s})\},\\ s & = & 0,\pm \,1,\pm \,2,\ldots \end{array}$$

This is the main result, showing the complex amplitude of the diffracted beam from an AMLPG and indicates that all the diffraction patterns forming over the individual diffraction orders are different.

It is worth noting that unlike the case of diffraction from the product of a given function and a linear grating in which the spectrum of the function is multiplied over different diffraction orders of the grating, here such behavior does not occur. The reason is that the structure of an AMLPG is not separable into a linear grating structure and another definite function.

Equation 17 can be considered as a summation over individual diffraction orders

18 $$U(r,\phi ,z)=\mathop{\sum }\limits_{s=-\infty }^{+\infty }\,{U}_{s}({r}_{s},{\phi }_{s},z\mathrm{).}$$

Similar to the diffraction of a plane wave from a radial phase grating^31^, here again we see that each of individual diffraction patterns has a radial form with a considerable structural complexity. All of the patterns are shape invariant under propagation. Therefore we consider each of them a “radial carpet beam”. In one of the next subsections we will show that for a specific value of the phase amplitude of an AMLPG having a given value of *l*, one of the individual diffraction patterns will get the same intensity distribution of the radial carpet beam produced directly in the diffraction of a plane wave from a radial phase grating with the same spokes number, *l*.

#### Relative rotation of the resulting patterns over ±*s* diffraction orders

We show that the diffraction patterns generated over a pair of diffraction orders ±*s* are similar in form but have a relative rotation with respect to each other.

For a given positive value of *s*, substituting *s* with −*s* in Eq. 17 we have the following equation:

19 $$\begin{array}{rcl}{U}_{-s}({r}_{-s},{\phi }_{-s},z) & = & {{\rm{e}}}^{ikz}{{\rm{e}}}^{i\alpha {r}^{2}}\{{(i)}^{-s}{J}_{-s}(\gamma ){J}_{0}(\,-\,s){e}^{-i\alpha {r}_{-s}^{2}}\\  &  & +\,\mathop{\sum }\limits_{q=1}^{+\infty }\,{(i)}^{-s}{(i)}^{-q(\frac{l}{2}-1)-1}{J}_{-s}(\gamma ){J}_{q}(\,-\,s){r}_{-s}(\frac{\pi }{\sqrt{\lambda z}}){e}^{\frac{-i\alpha {r}_{-s}^{2}}{2}}\\  &  & \times \,[{J}_{\frac{ql+1}{2}}(\frac{\alpha {r}_{-s}^{2}}{2})+i\,{J}_{\frac{ql-1}{2}}(\frac{\alpha {r}_{-s}^{2}}{2})]\,\cos (ql{\phi }_{-s})\},\end{array}$$

and using Eq. 7 we have

20 $${r}_{-s}=\sqrt{{r}^{2}+{(\frac{s\lambda z}{d})}^{2}+2\frac{s\lambda z}{d}r\,\cos \,\varphi },\,\& \,{\phi }_{-s}={\tan }^{-1}(\frac{r\,\sin \,\phi }{r\,\cos \,\phi +\frac{s\lambda z}{d}}).$$

Now we use Eq. 20 in Eq. 19 and we have

21 $$\begin{array}{rcl}{U}_{-s}({r}_{-s},{\phi }_{-s},z) & = & {{\rm{e}}}^{ikz}{(i)}^{s}\{{J}_{0}(s){J}_{s}(\gamma ){e}^{i\alpha ({r}^{2}-{r}_{-s}^{2})}\\  &  & +\,\mathop{\sum }\limits_{q=1}^{+\infty }\,{(i)}^{-q(\frac{l}{2}-1)-1}{J}_{q}(s){J}_{s}(\gamma ){r}_{-s}(\frac{\pi }{\sqrt{\lambda z}}){e}^{i\alpha ({r}^{2}-\frac{{r}_{-s}^{2}}{2})}\\  &  & \times \,[{J}_{\frac{ql+1}{2}}(\frac{\alpha {r}_{-s}^{2}}{2})+i\,{J}_{\frac{ql-1}{2}}(\frac{\alpha {r}_{-s}^{2}}{2})]\,\cos (ql({\phi }_{-s}-\frac{\pi }{l}))\},\end{array}$$

where we also used ${J}_{n}(\,-\,x)={(-1)}^{-n}{J}_{n}(x)$, $n\ge 0$ and $\cos (q\phi )={(-\mathrm{1)}}^{q}\,\cos (q\phi -q\pi )$, $q\in Z$.

Now by comparing Eqs 17 and 21 we can show the following result:

22 $${U}_{s}({r}_{s},{\phi }_{s},z)={U}_{-s}({r}_{-s},{\phi }_{-s}-\frac{\pi }{l},z).$$

This means that the resulting patterns over a pair of diffraction orders ±*s* are similar except there is a relative rotation with a value of $\frac{\pi }{l}$ between them.

### Controlled intensity sharing among different diffraction orders: Effect of *γ* on the resulting patterns

**Figure 2:**
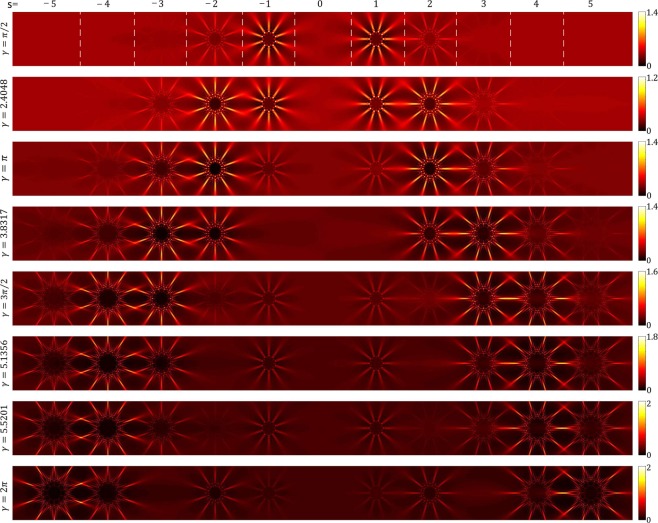
Controlled intensity sharing among the different diffraction orders of an AMLPG by adjusting the value of the phase amplitude, *γ*. Calculated intensity patterns of different diffracted beams in the diffraction of a plane wave from an AMLPG having $l=10$ and different values of *γ*: *π*/2, 2.4048, *π*, 3.8317, 3*π*/2, 5.1356, 5.5201, and 2*π* at $z=555\,{\rm{cm}}$. The intensity over the patterns is normalized to the intensity of the incident beam (for details see the color bars). The dashed white lines in the first row show the boundaries of the different diffraction orders.

Here we show that the intensity of an incident beam on an AMLPG can be divided among different diffraction orders with desired proportions. For an AMLPG, unlike the conventional gratings, the intensity share of the higher diffraction orders may be larger than the intensity on the lower diffraction orders, when given values are chosen for the phase amplitude of the grating, *γ*. Figure 2 shows the calculated diffracted intensity patterns for an AMLPG with $l=10$ spokes and different values of *γ* at a distance $z=555\,{\rm{cm}}$. By selecting the values of 2.4048, 3.8317, and 5.1356 for the arguments of *J*_0_, *J*_1_, and *J*_2_ their first zeros appear, respectively, and 5.5201 leads to the second zero of *J*_0_. As is apparent, the visibility of the patterns and intensity sharing among different diffraction orders depend to the value of *γ*.

- For $\gamma =\frac{\pi }{2}$ and $\gamma =2.4048$ the diffraction orders of $s=\pm \,1$ are more visible and they have the maximum values of the intensities between the diffraction orders.
- For $\gamma =\pi$ the diffraction orders for $s=\pm \,2$ are the dominant patterns.
- For $\gamma =3.8317$ and $\gamma =\frac{3\pi }{2}$ the diffraction orders for $s=\pm \,3$ are the dominant patterns and have the maximum share of the intensity.
- For $\gamma =5.1356$ and $\gamma =5.5201$ the diffraction orders for $s=\pm \,4$ share the maximum intensities.
- For $\gamma =2\pi$ the diffraction orders of $s=\pm \,5$ are more visible.

This means that by increasing the value of *γ* the energy of the incident light transfers to the higher diffraction orders. As is apparent, at larger radii, all individual diffraction patterns have the same number of spokes equal to the number of spokes of the AMLPG, but at smaller radii, the fine structure of the patterns strongly depends on the value of *s*. Also, it is seen that for larger values of *s*, the spoke patterns become needle-like and therefore the path of each needle-like pattern under propagation becomes a light sheet. These features might find some application in light sheet microscopy.

Now, we estimate the diffraction coefficients of an AMLPG by calculating the percentage of the incident power flows among different diffraction orders. This is done by calculating the ratios of the mean values of the intensities over the diffracted patterns to the mean value of the incident beam intensity. The mean value of the intensity over each of the individual patterns, separated by the dashed lines in the first row of Fig. 2, was calculated. Table 1 shows the results of the intensity sharing between different orders for some selected values of *γ*.

**Table 1:** Calculated ratios of the mean intensity of the diffraction order to the mean intensity of the incident beam for the diffraction patterns illustrated in Fig. 2.

s=	−5	−4	−3	−2	−1	0	1	2	3	4	5	$\tfrac{{\sum }_{{\boldsymbol{s}}=-{\bf{5}}}^{+{\bf{5}}}\,{\bar{{\boldsymbol{I}}}}_{{\boldsymbol{s}}}}{{\bar{{\boldsymbol{I}}}}_{{\boldsymbol{i}}}}$%
$\tfrac{{\bar{{\boldsymbol{I}}}}_{{\boldsymbol{s}}}}{{\bar{{\boldsymbol{I}}}}_{{\boldsymbol{i}}}}$	$\tfrac{{\bar{{\boldsymbol{I}}}}_{-{\bf{5}}}}{{\bar{{\boldsymbol{I}}}}_{{\boldsymbol{i}}}}$%	$\tfrac{{\bar{{\boldsymbol{I}}}}_{-{\bf{4}}}}{{\bar{{\boldsymbol{I}}}}_{{\boldsymbol{i}}}}$%	$\tfrac{{\bar{{\boldsymbol{I}}}}_{-{\bf{3}}}}{{\bar{{\boldsymbol{I}}}}_{{\boldsymbol{i}}}}$%	$\tfrac{{\bar{{\boldsymbol{I}}}}_{-{\bf{2}}}}{{\bar{{\boldsymbol{I}}}}_{{\boldsymbol{i}}}}$%	$\tfrac{{\bar{{\boldsymbol{I}}}}_{-{\bf{1}}}}{{\bar{{\boldsymbol{I}}}}_{{\boldsymbol{i}}}}$%	$\tfrac{{\bar{{\boldsymbol{I}}}}_{{\bf{0}}}}{{\bar{{\boldsymbol{I}}}}_{{\boldsymbol{i}}}}$%	$\tfrac{{\bar{{\boldsymbol{I}}}}_{{\bf{1}}}}{{\bar{{\boldsymbol{I}}}}_{{\boldsymbol{i}}}}$%	$\tfrac{{\bar{{\boldsymbol{I}}}}_{{\bf{2}}}}{{\bar{{\boldsymbol{I}}}}_{{\boldsymbol{i}}}}$%	$\tfrac{{\bar{{\boldsymbol{I}}}}_{{\bf{3}}}}{{\bar{{\boldsymbol{I}}}}_{{\boldsymbol{i}}}}$%	$\tfrac{{\bar{{\boldsymbol{I}}}}_{{\bf{4}}}}{{\bar{{\boldsymbol{I}}}}_{{\boldsymbol{i}}}}$%	$\tfrac{{\bar{{\boldsymbol{I}}}}_{{\bf{5}}}}{{\bar{{\boldsymbol{I}}}}_{{\boldsymbol{i}}}}$%	
*γ*=												
*π*/2	0.00	0.02	0.47	6.21	32.07	22.28	32.19	6.25	0.48	0.02	0.00	99.99
2.4048	0.02	0.42	3.94	18.58	26.90	0.00	27.00	18.70	3.98	0.42	0.02	99.98
*π*	0.27	2.28	11.07	23.49	8.09	9.26	8.12	23.64	11.17	2.30	0.27	99.96
3.8317	1.27	6.51	17.60	16.17	0.00	16.22	0.00	16.27	17.75	6.55	1.29	99.63
3*π*/2	4.93	13.66	16.40	2.13	7.92	7.07	7.95	2.15	16.54	13.77	5.01	97.53
5.1356	7.68	15.68	11.48	0.00	11.52	1.75	11.56	0.00	11.59	15.80	7.82	94.88
5.5201	10.34	15.62	6.27	1.51	11.56	0.00	11.60	1.52	6.32	15.73	10.52	90.99
2*π*	13.77	9.92	0.08	8.26	4.50	4.85	4.52	8.31	0.09	10.00	14.01	78.31

The red numbers show the maximum values of the intensity sharing in each row. By increasing the value of *γ*, the maximum values shift toward the higher orders.

Using Eq. 17, we can directly calculate the diffraction coefficients of an AMLPG as the ratio of the power of each order of diffraction *P*_*s*_ to the power of the incident beam *P*_*i*_

23 $${P}_{s,i}=\frac{{P}_{s}}{{P}_{i}}=\frac{{\int }_{{A}_{s}}\,{I}_{s}({r}_{s},{\phi }_{s},z)\,dA}{{\int }_{{A}_{i}}\,{I}_{i}(x,y,z)\,dA}=\frac{{\int }_{{A}_{s}}\,{u}_{s}({r}_{s},{\phi }_{s},z){u}_{s}^{\ast }({r}_{s},{\phi }_{s},z)\,dA}{{I}_{i}(x,y,z){A}_{i}}=\frac{{\bar{I}}_{s}}{{\bar{I}}_{i}},$$

where *I*_*i*_ and *I*_*s*_ are the intensity values over the incident beam and the diffracted beam of order *s*, respectively. Their mean values over the corresponding areas are given by ${\bar{I}}_{i}={I}_{i}$ and ${\bar{I}}_{s}=\frac{{\int }_{{A}_{s}}\,{u}_{s}({r}_{s},{\phi }_{s},z){u}_{s}^{\ast }({r}_{s},{\phi }_{s},z)\,dA}{{A}_{s}}$, respectively. The same results in Table 1 were derived using Eq. 23.

The following results in Table 1 are worth noting:

- For $\gamma =\frac{\pi }{2}$ and $\gamma =2.4048$, the diffraction orders with $s=\pm \,1$ have the maximum values of intensity sharing of 32% and 27%, respectively.
- For $\gamma =\pi$ the diffraction orders with $s=\pm \,2$, have the maximum values of intensity sharing of 23.5%.
- For $\gamma =3.8317$ and $\gamma =\frac{3\pi }{2}$, the diffraction orders with $s=\pm \,3$ have the maximum values of intensity sharing of 17.6% and 16.4%, respectively.
- For $\gamma =5.1356$ and $\gamma =5.5201$, the diffraction orders with $s=\pm \,4$ have the maximum values of intensity sharing of 15.7% and 15.6%, respectively.
- For $\gamma =2\pi$, the diffraction orders with $s=\pm \,5$ have the maximum values of intensity sharing of 13.8%.
- For the values of $\gamma =2.4048$ and $\gamma =5.5201$ the intensity sharing of the DC terms, with $s=0$, are zero. These values of *γ* correspond to the first and second zeros of *J*_0_.
- For the values of $\gamma =3.8317$ and $\gamma =5.1356$, the intensity sharing of the $s=\pm \,1$ and $s=\pm \,2$ terms are zero. The value of $\gamma =3.8317$ corresponds to the first zero of *J*_1_ and the value of $\gamma =5.1356$ corresponds to the first zero of *J*_2_.

### Comparison of the diffraction patterns of an AMLPG and a radial phase grating

Here we compare the diffraction pattern of a plane wave diffracted directly from a radial phase grating^31^ with the diffraction pattern of an AMLPG. The diffracted light field in the diffraction of a plane wave from a radial phase grating having a sinusoidal transmission function can be written as^31^

24 $$\begin{array}{rcl}\psi (r,\phi ,z) & = & {{\rm{e}}}^{ikz}\{{J}_{0}(\gamma )+\mathop{\sum }\limits_{q=1}^{+\infty }\,{(i)}^{-(\frac{l}{2}-1)q-1}{J}_{q}(\gamma )r(\frac{\pi }{\sqrt{\lambda z}})\\  &  & \times \,{e}^{\frac{-i\alpha {r}^{2}}{2}}[{J}_{\frac{ql+1}{2}}(\frac{\alpha {r}^{2}}{2})+i\,{J}_{\frac{ql-1}{2}}(\frac{\alpha {r}^{2}}{2})]\,\cos (ql\phi )\}.\end{array}$$

Comparing Eqs 17 and 24, we see that if the following relations are established

25 $$\begin{array}{rcl}{J}_{0}(\gamma ) & = & {(i)}^{s}{J}_{0}(s){J}_{s}(\gamma ),\\ {J}_{q}(\gamma ) & = & {(i)}^{s}{J}_{q}(s){J}_{s}(\gamma ),\end{array}$$

the two equations will be the same. It is apparent that this is the case for $\gamma =s$. This means that the diffraction pattern from a radial phase grating, known as the “radial carpet beam”^31^, and the *s*–th diffracted order of an AMLPG are similar when the phase amplitude and spokes number of the AMLPG are equal to the spokes number of the radial phase grating.

**Figure 3:**
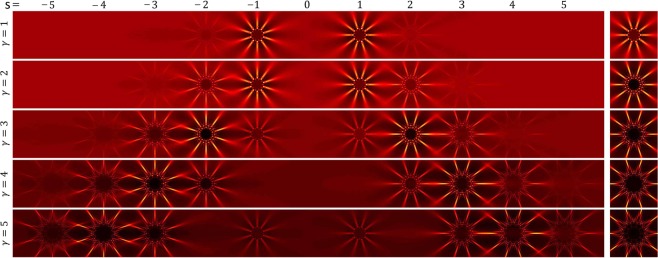
Comparing the diffraction pattens of an AMLPG and a radial phase grating having the same number of spokes. Left column, calculated diffracted intensity patterns obtained in the diffraction of a plane wave from an AMLPG with $l=10$ at $z=555\,{\rm{cm}}$ for *γ* equal to 1, 2, 3, 4, and 5. Right column, calculated diffracted intensity patterns obtained in the diffraction of a plane wave from a radial grating with $l=10$ at $z=555\,{\rm{cm}}$ for *γ* equal to 1, 2, 3, 4, and 5^31^. In each row, for $s=\gamma$, the *s*–th diffraction order of the AMLPG has the maximum value of intensity between all the diffraction orders and its diffraction pattern and the illustrated radial carpet pattern at the right column has the same form. The intensity over the patterns is normalized to the intensity of the incident beam.

In Fig. 3 the calculated diffraction pattern (or equally the whole spectrum) of an AMLPG (left column) and the diffraction pattern of a plane wave diffracted directly from a radial phase grating (right column) are illustrated. As it is seen for $s=\gamma$ we have ${U}_{s}{U}_{s}^{\ast }=\psi {\psi }^{\ast }$ and the *s*–th diffraction order has the maximum value of intensity between all the diffraction orders. From Figs 2 and 3, it is also seen that by increasing the value of *γ*, the higher diffraction orders get a larger fraction of the energy of the incident beam.

Since the value of intensity at different diffraction orders can be adjusted with the value of the grating phase amplitude, *γ*, this feature can be used in optical switching.

### Replication of the spectrum of an AMLPG by an SLM structure

Here we investigate the replication of the spectrum of an AMLPG by the SLM structure under diffraction. First we consider the diffraction of a plane wave from an SLM when a secondary structure is not embedded in it.

Assume that the structure of the SLM is a rectilinear 2D grating with the following transmission:

26 $$f(x,y)={f}_{x}(x){f}_{y}(y),$$

where *f*_*x*_ and *f*_*y*_ show two one dimensional (1D) linear gratings placed together at right angles. The Fourier transform of $f(x,y)$, apart from a multiplicative factor, is given by^36^

27 $$\begin{array}{rcl}F(\nu ,\eta ) & = & {F}_{x}(\nu ) \circledast {F}_{y}(\eta )\\  & = & \delta (\eta )\,\mathop{\sum }\limits_{m=0}^{+\infty }\,{B}_{m}\delta (\nu -\frac{m}{2{{\rm{\Lambda }}}_{x}}) \circledast \delta (\nu )\,\mathop{\sum }\limits_{n=0}^{+\infty }\,{B}_{n}\delta (\eta -\frac{n}{2{{\rm{\Lambda }}}_{y}}),\end{array}$$

where *δ* is the delta function, *B*_*m*_ and *B*_*n*_ are the Fourier coefficients, ${F}_{x}(\nu )$ and ${F}_{y}(\eta )$ are the 1D Fourier transforms of *f*_*x*_ and *f*_*y*_, respectively, and $\circledast$ is the convolution symbol.

Now suppose that the structure of an AMLPG with a transmission function of $T(\rho ,\theta )$ is imposed on the SLM and a plane wave is diffracted by it. In this case the light field immediately after the SLM is given by the multiplication of $f(x,y)$ and $T(\rho ,\theta )$, and the resulting diffraction pattern or equally the corresponding spatial spectrum is obtained as

28 $${U}_{total}(r,\phi )=F(\nu ,\eta ) \circledast U(r,\phi \mathrm{).}$$

The diffracted light field distribution or equally the spectrum of the AMLPG is replicated by the impulses of the SLM. The replicated spectrum by the $(m,n)$ impulse of the SLM can be written as

29 $${U}_{m,n}(r,\phi )={F}_{m,n}(\nu ,\eta ) \circledast U(r,\phi \mathrm{).}$$

This means that the spectrum of an AMLPG is replicated by each of the diffraction impulses of the SLM. Using Eqs 18 and 27 in Eq. 29, the distribution of the diffracted light field corresponding to the $(m,n)$ impulse of the SLM after a propagation distance of *z* is given by

30 $${U}_{m,n}({r}_{m,n},{\phi }_{m,n},z)=\mathop{\sum }\limits_{s=-\infty }^{+\infty }\,{U}_{m,n,s}({r}_{m,n,s},{\phi }_{m,n,s},z),$$

where

31 $$\begin{array}{rcl}{U}_{m,n,s}({r}_{m,n,s},{\phi }_{m,n,s},z) & = & {{\rm{e}}}^{ikz}{{\rm{e}}}^{i\alpha {r}^{2}}\{{(i)}^{s}{J}_{s}(\gamma ){J}_{0}(s){e}^{-i\alpha {r}_{m,n,s}^{2}}\\  &  & +\,\mathop{\sum }\limits_{q=1}^{+\infty }\,{(i)}^{s-1}{(i)}^{-q(\frac{l}{2}-1)}{J}_{s}(\gamma ){J}_{q}(s){r}_{m,n,s}(\frac{\pi }{\sqrt{\lambda z}}){e}^{\frac{-i\alpha {r}_{m,n,s}^{2}}{2}}\\  &  & \times \,[{J}_{\frac{ql+1}{2}}(\frac{\alpha {r}_{m,n,s}^{2}}{2})+i\,{J}_{\frac{ql-1}{2}}(\frac{\alpha {r}_{m,n,s}^{2}}{2})]\,\cos (ql{\phi }_{m,n,s})\},\end{array}$$

in which ${r}_{m,n,s}=\sqrt{{({r}_{s}\cos {\phi }_{s}+m\frac{\lambda z}{{{\rm{\Lambda }}}_{x}})}^{2}+{({r}_{s}\sin {\phi }_{s}+n\frac{\lambda z}{{{\rm{\Lambda }}}_{y}})}^{2}}$ and ${\phi }_{m,n,s}={\tan }^{-1}(\frac{{r}_{s}\,\sin \,{\phi }_{s}+n\frac{\lambda z}{{{\rm{\Lambda }}}_{y}}}{{r}_{s}\,\cos \,{\phi }_{s}+m\frac{\lambda z}{{{\rm{\Lambda }}}_{x}}})$.

The patterns of various radial carpet beams over different diffraction orders of the AMLPG with controlled intensity sharing among the generated beams are replicated over the SLM diffraction orders. Each of the generated radial carpet beams is given by

32 $${U}_{m,n,s}(r,\phi )={F}_{m,n}(\nu ,\eta ) \circledast {U}_{s}(r,\phi );s\in Z,$$

where *s* shows the order of diffraction of the AMLPG imposed on the SLM.

## Discussion

We show that by adding an azimuthally periodic term into the argument of a linear phase grating, say $\cos (l\theta )$ in Eq. 1, and by adjusting the value of *γ*, one can control the intensity sharing between different diffracted beams (see Figs 2 and 3). The theoretical perditions show that, in order to have maximum share of energy on a higher order diffraction pattern, we need to use an SLM with a large value of phase variation. The proposed method for controlling the intensity sharing between different diffraction orders can be implemented with the aid of other additional phase terms in Eq. 1. For example, by replacing $\cos (l\theta )$ with the phase function of a zone plate $\cos (\frac{\pi {\rho }^{2}}{s})$, where *s* is the zone plate constant, at given propagation distances over the different diffraction patterns focused beams with different values of intensities will be produced. This feature can be used for optical switching. Another example is the phase function of a defected zone plate $\cos (l\theta +\frac{\pi {\rho }^{2}}{s})$. The results of these studies will appear elsewhere.

## Methods

In this section we present experimental work that verifies the above theoretical results.

### Experimental generation of the various radial carpet beams

We used a conventional SLM extracted from a video projector (LCD projector KM3 MOD. NO. X50) to provide the desired AMLPGs. Maximum amplitude of the phase modulation was limited to $\gamma =\pi /2\,{\rm{rad}}$ shown in Fig. 1. In the experiments, the whole area of the SLM was illuminated with a plane wave which was the second harmonic of an Nd:YAG diode–pumped laser beam having a wavelength of $\lambda =532\,{\rm{nm}}$. The active area of the SLM was 11 mm × 15 mm.

An AMLPG was imposed on the SLM and the collimated wavefront of the laser beam propagated through it. At different distances from the SLM, the diffracted patterns were recorded with a camera (Nikon D7200). We recorded the diffraction patterns by two methods. In some parts of the experiments, the diffraction patterns were directly formed over the active area of the camera by removing the imaging lens of the camera. Also in some of the experiments, the desired diffraction patterns were formed on a diffuser and then their patterns were imaged by the imaging lens of the camera on its active area. In the latter case, a magnification in the size of the images appeared. The active image area of the camera was 23.4 mm × 15.6 mm.

**Figure 4:**
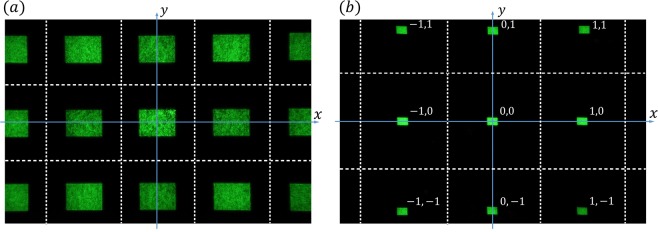
Experimentally recorded diffraction pattern at a propagation distance of (**a**) $z=77\,{\rm{cm}}$ and (**b**) $z=350\,{\rm{cm}}$, in the diffraction of a plane wave from an SLM when it experiences a uniform phase map. The real size of the recorded rectangular patterns is 11 mm × 15 mm. The pairs of numbers correspond to the diffraction orders of the SLM’s impulses. The dashed white lines are used to distinguish the areas of different diffraction orders.

Figure 4(a) shows an experimentally recorded diffraction pattern of a plane wave from the SLM when a uniform phase map is imposed on it. A diffuser was placed at $z=77\,{\rm{cm}}$ and the pattern was imaged by the camera. Since the SLM has a two dimensional periodic structure, each of the observed rectangular patterns in Fig. 4(a) corresponds to one of the diffraction orders of the SLM’s main structure. Figure 4(b) shows the same pattern after a propagation distance of $z=350\,{\rm{cm}}$. In Fig. 4(b), pairs of numbers show the numbers of diffraction orders in the horizontal (*x*) and vertical (*y*) directions corresponding to the SLM’s main structure.

**Figure 5:**
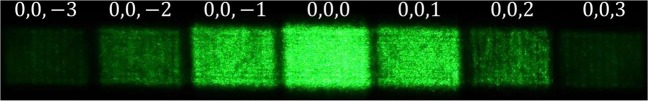
Diffraction pattern of a plane wave from an SLM at $z=350\,{\rm{cm}}$ when a 1D linear phase grating with a sinusoidal profile in the *x* direction and a period of 0.11 mm is imposed on the SLM. Here $\gamma =\pi /2$. This pattern corresponds to the (0,0) diffraction order of the SLM and was formed on a diffuser and imaged by camera.

Figure 5 shows the diffraction pattern of a plane wave from the SLM when a 1D linear phase grating with a sinusoidal profile in the *x* direction and a period of 0.11 mm is imposed on it. The transmittance of a linear phase grating with a sinusoidal profile is given by Eq. 1 when *cos(lθ)* = 0. The sets of numbers indicate the orders of the diffraction from the SLM structure and the linear phase grating, $(m,n,s)$.

**Figure 6:**
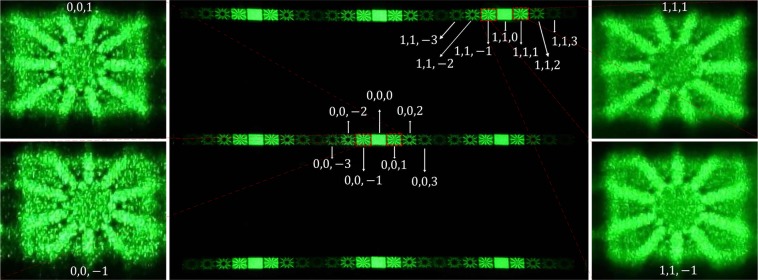
Central pattern: The diffraction pattern of a plane wave from an SLM with $\gamma =\pi /2$ when an AMLPG is imposed on the SLM. Here a diffuser is placed at $z=350\,{\rm{cm}}$ and the diffraction pattern is imaged by camera. For the radial phase structure $l=10$ and for the linear phase grating $d=0.11\,{\rm{mm}}$. The diffraction patterns of four typical diffraction orders are enlarged in the first and third columns.

In Fig. 6, second column, the central area of the diffraction pattern of a plane wave from an SLM with $\gamma =\pi /2$ is shown when an AMLPG is imposed on it. In the experiment, a diffuser was placed at $z=350\,{\rm{cm}}$ and the central area of the diffraction pattern imaged by the camera. The radial phase structure with $l=10$ is imposed on the structure of a linear phase grating with a period of $d=0.11\,{\rm{mm}}$ in the *x* direction (see Eq. 1). For better illustration of the results, four typical diffraction orders are enlarged in the first and third columns in Fig. 6. Each of the illustrated diffraction patterns is determined by the corresponding diffraction order $(m,n,s)$. As is apparent, the intensity profiles for two individual diffraction patterns are the same when their order numbers, *s*, are the same. For two given individual diffraction patterns with orders $(m,n,s)$ and $(m,n,-\,s)$, the patterns are rotated at an angle $\frac{\pi }{l}$ with respect to each other.

**Figure 7:**
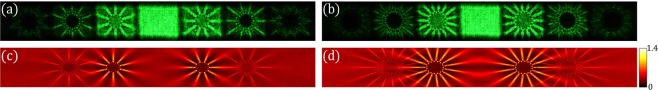
(**a**, **b**) are experimental diffraction patterns of two AMLPGs with $l=10$ and $l=15$ at $z=350\,{\rm{cm}}$, respectively. These patterns are generated over the (0,0) diffraction order of the SLM. Here $\gamma =\pi /2$. (**c**, **d**) are the corresponding theoretical patterns. The intensity over the simulated patterns is normalized to the intensity of the incident beam, and for this reason a color bar is used for (**c**, **d**).

Figure 7(a,b) show the experimental diffraction patterns corresponding to the $(0,0)$ diffraction order of the SLM when two AMLPGs with $l=10$ and $l=15$ were imposed on it, respectively. Here again, a diffuser was placed at $z=350\,{\rm{cm}}$ and the central diffraction order of the SLM with order number (0,0) was imaged by the camera. In Fig. 7(c,d) the corresponding theoretically produced patterns are illustrated. As it is seen, when the value of *l* is odd, the intensity patterns of ${I}_{(m,n,s)}$ and ${I}_{(m,n,-s)}$ are mirrors of each other relative to the plane of $s=0$ order.

**Figure 8:**
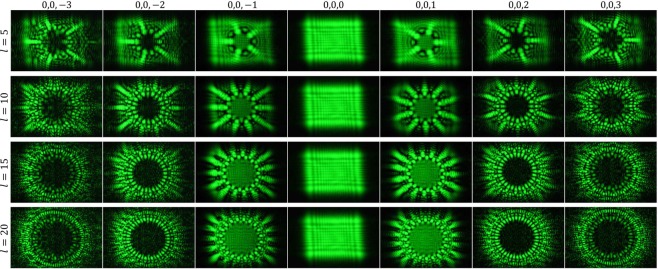
Experimentally generated diffraction patterns of four AMLPGs with $l=5$, $l=10$, $l=15$, $l=20$, and $\gamma =\pi /2$. Each of the individual patterns was formed directly on the active area of the camera at a distance of $z=555\,{\rm{cm}}$. A set of low contrast crossed linear fringes appear over the central patterns (0,0,0). These fringes are the edge diffraction patterns of the SLM window.

In Fig. 8 experimentally recorded diffraction patterns are shown for four AMLPGs having spokes numbers of 5, 10, 15, and 20, respectively. Here, each of the individual patterns was recorded directly over the active area of the camera by removing its imaging lens at distance $z=555\,{\rm{cm}}$.

## Conclusion

In this work, we introduced a new kind of phase grating with controlled intensity among different diffraction orders. We constructed an AMLPG by adding an azimuthal periodic dependency to the argument of the transmission function of a linear phase grating having a sinusoidal profile. Generation of diverse radial carpet beams was investigated over different diffraction orders of an AMLPG with controlled predominant diffraction order. A detailed theoretical analysis was reported and its experimental verification was presented by generating diverse radial carpet beams with controlled shift of intensity in the illumination of an AMLPG with a spatially coherent light beam. We specified diverse radial carpet beams produced over different diffraction orders of the host linear phase grating. It was shown that all the diffraction patterns are different and only the pairs of positive and negative orders with the same order numbers are similar except that there is a relative rotation between them. The diffraction grating introduced with controlled intensity sharing among different diffraction orders might find wide applications in many areas of optics such as optical switches. Also, the radial carpet beams produced over different diffraction orders might find applications in light sheet microscopy.
